# To Preserve Blood Corpuscles for Microscopic Examination

**Published:** 1889-04

**Authors:** V. A. Latham


### To Preserve Blood Corpuscles for Microscopic Examination.

V. A. LATHAM.

Spread a thin film of blood on a covered glass and gently dry, inverted for thirty minutes or more into a covered capsule containing a half saturated solution of apranin in absolute alcohol. The stain is then washed off by a stream of distilled water. Dry the specimen thoroughly and mount, either in chloroform balsam liquified by heat or thinned by turpentine. In specimens I have of Trap blood, it shows the nuclei dark pink, and the rest of the corpuscles lightly tinted. In human blood, they become a clear pink color, and do not fade.—*Jour. Anat. and Physiol.*

